# Performance Evaluation of Regression Models for the Prediction of the COVID-19 Reproduction Rate

**DOI:** 10.3389/fpubh.2021.729795

**Published:** 2021-09-14

**Authors:** Jayakumar Kaliappan, Kathiravan Srinivasan, Saeed Mian Qaisar, Karpagam Sundararajan, Chuan-Yu Chang, Suganthan C

**Affiliations:** ^1^School of Computer Science and Engineering, Vellore Institute of Technology, Vellore, India; ^2^Electrical and Computer Engineering Department, Effat University, Jeddah, Saudi Arabia; ^3^School of Information Technology and Engineering, Vellore Institute of Technology, Vellore, India; ^4^Department of Computer Science and Information Engineering, National Yunlin University of Science and Technology, Douliu, Taiwan; ^5^School of Social Sciences and Languages, Vellore Institute of Technology, Vellore, India

**Keywords:** COVID-19, feature selection, machine learning, prediction error, reproduction rate prediction, regression

## Abstract

This paper aims to evaluate the performance of multiple non-linear regression techniques, such as support-vector regression (SVR), k-nearest neighbor (KNN), Random Forest Regressor, Gradient Boosting, and XGBOOST for COVID-19 reproduction rate prediction and to study the impact of feature selection algorithms and hyperparameter tuning on prediction. Sixteen features (for example, Total_cases_per_million and Total_deaths_per_million) related to significant factors, such as testing, death, positivity rate, active cases, stringency index, and population density are considered for the COVID-19 reproduction rate prediction. These 16 features are ranked using Random Forest, Gradient Boosting, and XGBOOST feature selection algorithms. Seven features are selected from the 16 features according to the ranks assigned by most of the above mentioned feature-selection algorithms. Predictions by historical statistical models are based solely on the predicted feature and the assumption that future instances resemble past occurrences. However, techniques, such as Random Forest, XGBOOST, Gradient Boosting, KNN, and SVR considered the influence of other significant features for predicting the result. The performance of reproduction rate prediction is measured by mean absolute error (MAE), mean squared error (MSE), root mean squared error (RMSE), R-Squared, relative absolute error (RAE), and root relative squared error (RRSE) metrics. The performances of algorithms with and without feature selection are similar, but a remarkable difference is seen with hyperparameter tuning. The results suggest that the reproduction rate is highly dependent on many features, and the prediction should not be based solely upon past values. In the case without hyperparameter tuning, the minimum value of RAE is 0.117315935 with feature selection and 0.0968989 without feature selection, respectively. The KNN attains a low MAE value of 0.0008 and performs well without feature selection and with hyperparameter tuning. The results show that predictions performed using all features and hyperparameter tuning is more accurate than predictions performed using selected features.

## Introduction

The world has witnessed several deadly diseases at different times. In the year 2020, the world suffered a serious pandemic that took away many lives (1). The coronavirus disease (COVID-19) is a disease that started as an epidemic and evolved into a pandemic. The disease was first discovered in late December 2019 in Wuhan, China (2). The virus responsible for causing the disease is the severe acute respiratory syndrome coronavirus 2 (SARS-CoV-2), which is highly contagious and causes severe respiratory issues. The virus rapidly spread across the world and affected 223 countries, infected more than 9.3 × 10^7^ people, and took over 2 × 10^6^ human lives, according to the WHO report in January 2021 (3). As a result, scientists and epidemiologists worldwide are investigating the virus to reduce its impact on human lives.

The coronavirus is named after the word, “coronation” since the spikes on the surface of the virus resemble a crown. This virus was believed to be an animal virus in 2002. The SARS-CoV is mostly found in bats and transmitted to other animals, such as cats. The first human-infected coronavirus case was reported in 2003 in Guangdong province in the south of China (4).

Knowledge of the immune system of our body is required to understand the mechanisms of the COVID-19 or any other viral infections. Viruses are microorganisms that make our body cells their hosts for replication and multiplication. The immune system of our body is activated by the entry of the virus and identifies the virus as an alien body for destruction. After attacking and killing the viruses, the immune system “remembers” the virus and launches the same protective measures when the virus enters again. Viruses are capable of fast evolutions. They evolve to new shapes or mechanisms to survive in the changing environment.

Viral infections often affect people with weak immune systems. The elderly, children, and people with medical conditions are prone to the attack of novel viruses. The virus can be deadly and threatening to the senior population, especially the elderly with chronic medical conditions.

The SARS-CoV-2 is transmitted *via* respiratory droplets expelled by sneezing, coughing, or talking. The virus can also be contracted by touching a contaminated surface. One significant property of the SARS-CoV-2 is its capacity to survive on various surfaces for up to 9 days at room temperature, which facilitates its rapid transmission (5). Acute Respiratory Disease Syndrome is caused predominantly by this virus and often leads to multiple organ dysfunctions, resulting in physiological deterioration and even death of the infected persons (6).

This study is intended to predict the rate of reproduction of the deadly SARS-CoV-2. The reproduction rate (R_o_) is an important parameter to predict the spread of a disease in a pandemic situation. The R_o_ value indicates the transmissibility of a virus through the average number of new infections caused by an infectious person in a naïve population. The value of R_o_ <1 indicates that the infection would die out. On the other hand, if the value is >1, the spread of the disease would increase. For example, a reproduction rate of 18 indicates that a single infected individual can potentially infect 18 more individuals. The reproduction rate is needed to determine whether the disease is under control or turning into an epidemic.

There are many standard methods to predict the reproduction rate. The XGBoost is an optimal Gradient Boosting algorithm with tree pruning, parallel processing, missing value handling, and by the efficient use of hardware resources and regularization to avoid overfitting and bias. The XGBoost has faster computational times (7) in all types of environments. The XGBoost is an improvement on the Gradient Boosting algorithm. Training models with an XGBoost iterative boosting approach remove errors at preceding boosting trees in the following iterations (8). Support vector regression (SVR) was based on the Vapnik–Chervonenkis (VC) theory. It is used when the output is a continuous numerical variable. Support vectors are data points closest to the hyperplanes. The hyperplanes represent the decision boundaries. The Radial Basis Function is a commonly used kernel function. The use of a kernel function is to transform the data into a higher-dimensional space. The SVR and convolutional neural network (CNN) have been used to detect groundwater location, and comparative results showed that the SVR outperforms the CNN (9). In k-nearest neighbor (KNN), the outcome of the variable is determined by taking an average of the observations found in the same neighborhood. The KNN algorithm assigns a weight, “W” to the KNN and a weight of 0 to the others.

The performance of the machine learning algorithms depends on the hyperparameter values. The values for the hyperparameters can be assigned in three ways:

1) Based on default values given in the software packages.
2) Manually configured by the user.
3) Assigned by algorithms, such as the simple grid search, random search, Bayesian optimization, ANOVA approach, and bio-inspired optimization algorithms.

The process of identifying the most relevant features is referred to as “feature selection.” The three main advantages of feature selection are:

- Simplifying the interpretation of the model.
- Reducing the variance of the model to avoid overfitting.
- Reducing the computational cost (and time) for model training.

### Motivation

Artificial intelligence (AI) has been successful in many fields and facilitates our daily life in various ways (10–17). The reproduction rate prediction is crucial in successfully establishing public healthcare in the battle against COVID-19. The prediction of the reproduction rate is performed by using not just the past values but also by using the closely related factors. This work also investigates the impact of feature selection and hyperparameter tuning on the performance of non-linear machine learning techniques.

### Research Gap and Contribution

The reproduction rate is related to many factors, such as the average number of contacts a person has, number of days a person is infected, from the day of exposure to the disease, the number of active cases, values of stringency index, testing capacity and positivity, and so on. As a result, the reproduction rate, time curve, and future values cannot be satisfactorily estimated by the probability distribution functions alone (18).

Time series prediction models, such as the autoregressive integrated moving average (ARIMA), Gray Model, and Markov Chain models do not consider multiple factors in reproduction rate prediction. Autoregressive models assume that future values resemble the past. Mechanistic models based on the susceptible, exposed, infected, and recovered (SEIR) states framework or modified version of the framework use the time series data to hold the currently confirmed cases, removed cases (including recovered and deceased cases), and time-varying transmission rates. However, some factors are still not included, and there is no weighting for the factors (19).

So there arises a need to study the various factors acting on the reproduction rate and to prioritize it. Hence identifying the importance of various features (for example, Total_cases_per_million, Total_deaths_per_million) under factors like active cases, stringency index, testing capacity, and positivity are done using feature selection algorithms. Multiple regression uses several explanatory variables to predict the single response variable. The performance of the non-linear machine learning techniques, such as Random Forest, XGBOOST, Gradient Boosting, KNN, and SVR are used in reproduction rate prediction. The performance of these approaches for predicting the COVID-19 reproduction rate was measured using the evaluation metrics like mean absolute error (MAE), mean squared error (MSE), **r**oot mean squared **e**rror (RMSE), R-Squared, relative absolute **e**rror **(**RAE), and root relative squared error (RRSE). The influence of feature selection and hyperparameter tuning operation on their performance is also studied.

### Structure of the Paper

Section Introduction of the paper introduces the reproduction rate, feature selection, machine learning techniques and hyperparameters, and the motivation of this study. Section Related Works discusses the related works, as well as the identified research gap and the contributions. Section Materials and Methods describes the methods used in this work, including feature selection, hyperparameter tuning, and prediction and performance measurement. Section Results and Discussion discusses the experimental results. Finally, section Conclusion and Future Work provides the conclusion and future work.

## Related Works

Zivkovic et al. (20) proposed a hybridized machine learning, adaptive neuro-fuzzy inference system with enhanced beetle antennae search (BAS) swarm intelligence metaheuristics. The results showed that the system is a good prediction model in time series forecasting. The defects in the BAS algorithm were rectified using the Cauchy exploration strategy BAS (CESBAS) using the Cauchy mutation and three additional control parameters. The selection of optimum values for the adaptive network-based fuzzy inference system **(**ANFIS) parameters became an NP-hard optimization problem. The ANFIS parameters values were determined using the CESBAS metaheuristics algorithm. The performance metrics, such as RMSE, MAE, MAPE, RMRE, and R-Squared, were used to evaluate the outcomes on influenza datasets.

The research goal in Mojjada et al. (21) was to forecast the number of new COVID-19 cases, mortalities, and recoveries using various machine learning regression models, such as the lowest absolute and selective shrinking operator (LASSO), vector supports, such as short message service (SMS), and exponential smoking (ES) models. While the linear regression and LASSO models were more effective in estimating and verifying the death rate, the ES model provided the overall best results.

Farooq and Bazaz (22) used an artificial neural network (ANN) to forecast the COVID-19 based on an online incremental learning technique using an adaptive and non-intrusive analytical model. The COVID-19 data was updated every day, so online incremental learning was the best option for forecasting since there is no need to retrain or rebuild the model from scratch.

Milind et al. (23) discovered many factors behind the spread of the coronavirus, such as the relationship between the weather and the spread of COVID-19, growth rate, and mitigation. Support vector regression (SVR) was used to predict the transmission rate, epidemic end, and the spread of the coronavirus across regions, and to analyze the growth rates and the types of mitigation across countries. The Pearson coefficient was used in the correlation between the coronavirus and weather correlation coefficient. Weather factors, such as the wind speed, temperature, and humidity of Milan city in Italy and New York City in the United States were considered. The SVR is a non-parametric technique since it only depends on the kernel function, implying that there is no need to change the explanatory variables in constructing a non-linear model. The study also compared the performances of SVR, linear regression, and polynomial regression.

Chicco and Jurman (24) predicted the survival of patients who had heart failure based on the ejection fraction and serum creatinine level. A database of 299 patients collected in 2015 was used. Feature selection was performed, and the factors were ranked. The ejection fraction and serum creatinine levels were found to be highly relevant among the 13 selected features. As a result, the prediction model was built and executed based on these two factors.

Mortazavi et al. (25) investigated the capability of machine learning techniques when applied to a high dimensional and non-linear relationship. They predicted the readmission of patients hospitalized for heart failure. The prediction was performed with various machine learning techniques, such as Random Forest, Gradient Boosting, and Random Forest combined hierarchically with support vector machines (SVMs) or logistic regression (LR) and Poisson regression. The obtained results were tested against traditional LR methods. The model was evaluated using the receiver operating characteristics (ROC) curve (C statistic), the positive predictive value (PPV), sensitivity, specificity, and f-score. The ROC was found to be a good measure for model discrimination.

Balli (26) analyzed the COVID-19 data from Germany, the United States, and other parts of the world. Methods including the SVM, linear regression, multilayer perceptron, and Random Forest methods were used to model the COVID-19 data. The performances of the methods were compared using the RMSE, absolute percentage error (APE), and mean absolute percentage error (MAPE). Among the tested methods, the SVM outperformed all other methods in the COVID-19 data modeling and was successfully used to diagnose the behavior of cumulative COVID-19 data over time.

A system to handle the data with non-linear relationships and non-normal distribution was proposed by Kuo and Fu (27). A total of 52 input variables relating to confirmed cases, environment variables, country-dependent variables, community mobility variables, and time series variables were used in the study (27). The impact of population mobility had caused an increase in the number of infections over the weekend. This work served as a basis for researchers analyzing geographical characteristics, seasonality, as well as models, such as long short-term memory (LSTM), ARIMA, convolutional neural network (CNN), and so on.

The COVID Patient Detection System (CPDS) used by Shaban et al. (28) was designed using a Hybrid Feature Selection Method (HFSM) consisting of two stages, a fast selection stage (FS^2^) and an accurate selection stage (AS^2^). The FS^2^ used several filter methods, and the filtered features served as the initial population of the genetic algorithm, which was used as a wrapper method. An enhanced K-nearest neighbor (EKNN) classifier was used to solve the trapping problem. The most significant features from the chest CT images of patients were selected. The HFSM allowed the EKNN classifier to obtain rapid predictions with high accuracy. The proposed feature selection algorithm was compared with four recent feature selection techniques, and the proposed CPDS had achieved an accuracy of 96%.

Sujatha et al. (29) utilized the linear regression, multi-layer perceptron (MLP), and vector autoregression (VAR) models to foresee the spread of the COVID-19 using the COVID-19 Kaggle data. The correlations between the features of the dataset are crucial in finding the dependencies. The VAR model is a more suitable analysis model for multivariate time series. It is an m-equation, m- variable model where an individual variable is based on its current and past values. The MLP methods provide better predictions than the linear regression and VAR models.

Yang et al. (30) predicted the number of new confirmed cases using SEIR and AI methods. The authors used the probability of transmission, incubation rate, and the probability of recovery or death as factors in the predictions. New case predictions made by the AI method are more accurate than the SEIR predictions.

The Gradient Boosting Feature Selection (GBFS) algorithm learns the ensemble of regression trees to identify the non-linear relationship between features with ease. The classification error rates for the GBFS and Random Forest methods are the lowest, whereas the L1-regularized logistic regression (L1-LR) and Hilbert–Schmidt independence criterion (HSIC) Lasso methods have higher error rates (31).

The XGBOOST algorithm was applied to calculate the business risk by Wang (32). Several feature selection methods were used to find the redundant features. Two hyper-parameter optimization approaches were applied: random search (RS) and Bayesian tree-structured Parzen Estimator (TPE). The XGBOOST with hyper-parameter optimization performed well for business risk modeling.

Chintalapudi et al. (33) used the predicted reproduction rate to forecast the daily and the cumulative COVID-19 cases for the next 30 days in Marche, Italy. The probability-based prediction was performed with the maximum likelihood function. In the implementation, a simple linear regression method was used to fit the exponential growth of infected incidences over time, and the linear regression was applied over the incidence data. This study showed that the outbreak size and daily incidence are primarily dependent on the daily reproductive number.

Locatelli et al. (34) estimated the COVID-19 reproduction rate of Western Europe with the average from 15 countries. The authors used the generation interval, defined as the time needed for an infected person to infect another person and for reproduction rate estimation. The works by Zhang et al. (35) and by Srinivasu et al. (36), Panigrahi et al. (37, 38), Tamang (39), Chowdhary et al. (40), and Gaur et al. (41) demonstrated the efficacy of machine learning algorithms in various fields.

## Materials and Methods

**Figure 1:**
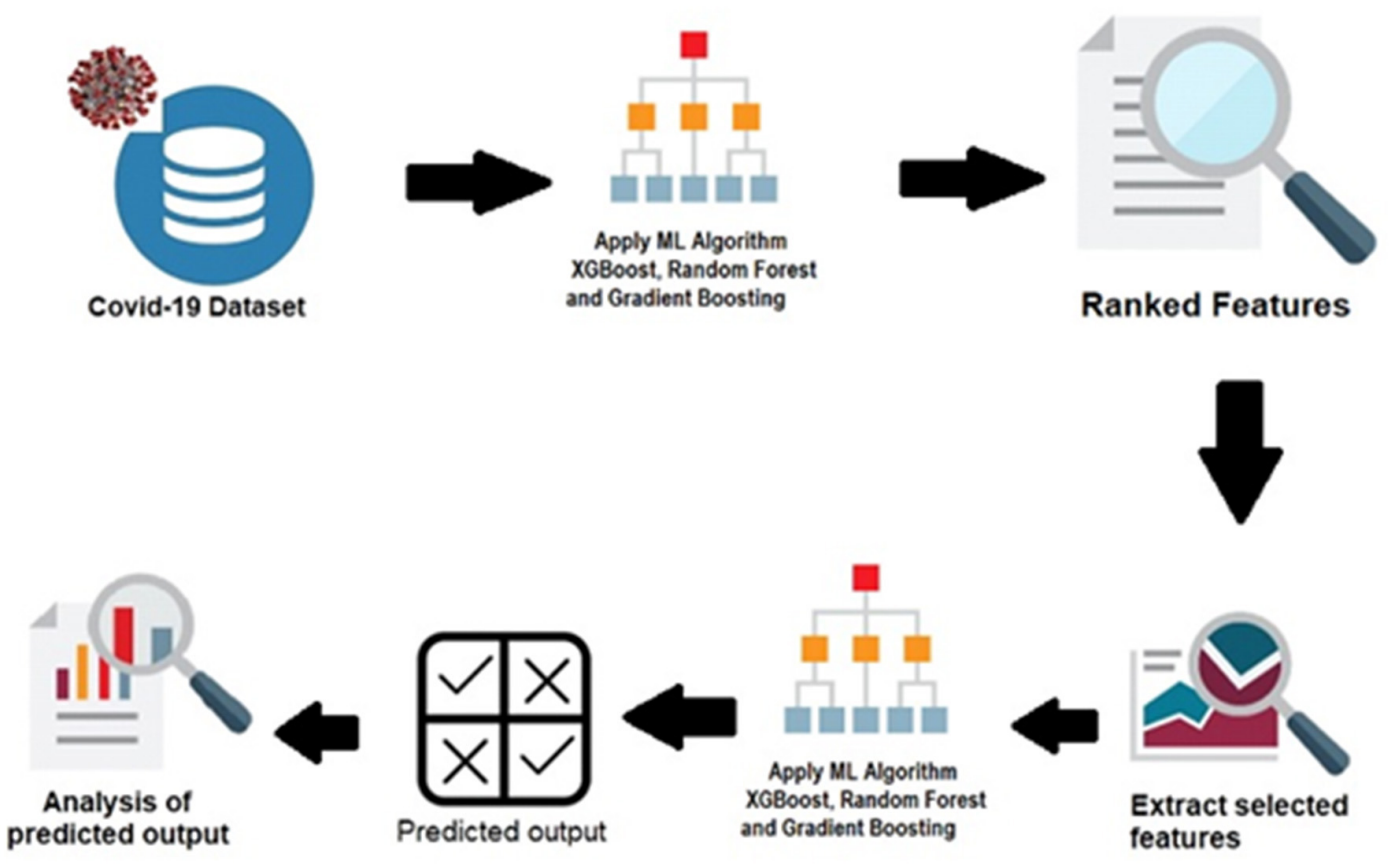
Proposed system architecture.

The spread of the COVID-19 depends on many factors. New factors influencing the spread of the disease are still being discovered, and the identification of predominant factors is crucial. The prediction of COVID-19 spread is highly related to the feature-reproduction rate. Data science can be applied to track the crucial features used for the prediction from any number of features. Traditional statistical approaches, such as the chi-square and Pearson correlation coefficient provide the importance of the features in relation to the other features. Feature selection reduces the overfitting and underfitting problems, computational cost, and time. The reproduction rate prediction is important since it is associated with the status of the COVID-19. Feature-ranking is performed using Random Forest regression, Gradient Boosting, and XGBoost. Seven factors are considered in this study: the total number of cases, number of new cases, total number of deaths, total number of cases per million, total number of deaths per million, total number of tests conducted per thousand, and the positive rate. The proposed system architecture is represented in Figure 1.

### Reproduction Rate

Newly occurring diseases can be detrimental for humans and other animals, whether the diseases are caused by a new pathogen or a modified form of an existing pathogen (19). In this work, the simple compartmental disease models and matrix methods are used to calculate the reproduction rate, R0.

### Feature Selection

Embedded filter-based feature selection methods, such as Random Forest, Gradient Boosting, and XGBoost, which take into account the regression process, are used in this work. The Random Forest approach is an embedded feature selection method in which hundreds of decision trees are constructed by extracting random observation values of random features. The training determines features that reduce impurity. The principle of Gradient Boosting, and XGBoost methods are used to boost the weak learners. Gradient Boosting strives to minimize the error between the predicted and the actual values. The XGBoost is an extreme Gradient Boosting algorithm. The XGBoost is the regularized form of Gradient Boosting (42). The XGBoost is fast with L1, L2 regularization and parallel computing. It delivers high performance since it works on the second partial derivatives of the loss function. The main highlight of the Random Forest algorithm lies in its ability to prevent overfitting and increase accuracy. The advantage of gradient boosting is its ability to tune many hyperparameters and loss functions.

### Parameter Settings

The experiments use multiple non-linear regression tree algorithms and the result is implemented in Python. For experiments without hyperparameter tuning, the default values in the SciKit library are used. For Random Forest regression, the parameter values are initialized as follows: n_estimators = 100, n_jobs = −1, oob_score = True, bootstrap = True, and random_state = 42. For XGBoost, the XGB Regressor method is used to fit the test data, and n_estimatorsis set to 100. The Gradient Boosting feature importance is calculated by setting the value of n_estimators to 500, max_depth to 4, min_samples_split to 5, learning_rate to 0.01, and loss as ls. For the KNN algorithm, the lower error rate is achieved when the K value equals 7. For the SVR, the radial basis function kernel is used with degree = 3 and gamma = scale. For experiments with hyperparameter tuning, grid search and randomized approaches are used. A grid search exhaustively tests all possible combinations of the specified hyperparameter values for an estimator. In a randomized search, the model selects the combinations randomly. Both approaches are very effective ways of tuning the parameters to increase the generalizability of the model. The GridSearchCV method of sklearn tunes the hyperparameters of the SVR, KNN, XGBoost, and Gradient Boosting approaches. The randomized search CV function is used for the hyperparameter tuning of Random Forest Regressor.

### Dataset

The dataset was taken from the website “https://github.com/owid/covid-19-data/tree/master/public/data” (43). A total of 16 fields were used for the study of reproduction rate. They are Total_cases, New_cases, Total_deaths, New_deaths, Total_cases_per_million, New_cases_per_million, Total_deaths_per_million, New_deaths_per_million, New_tests, Total_tests, Total_tests_per_thousand, New_tests_per_thousand, Positive_rate, Tests_per_case, Stringency_index, Population_density. Records from April 1, 2020, to November 30, 2020, are used as training data (244 records/day). Records from December 1, 2020, to March 10, 2021, are used as testing data (100 records/day).

### Performance Metrics

Numerous machine learning (ML)-based predictive modeling techniques are used in the COVID-19 predictions. Therefore, there is a need to measure the performance of each model and its prediction accuracy. The metrics used to assess the effectiveness of the model in predicting the outcome are very important since they influence the conclusion. The performance metrics to identify the error rate between the predicted and observed values are as follows:

- Root mean square error (RMSE)
- Mean absolute error (MAE)
- Determination coefficient (*R*^2^)
- Relative absolute error (RAE)
- Root relative squared error (RRSE)

#### Mean Absolute Error

The mean absolute error measures the sum of the absolute differences between the predicted output and the actual output. One cannot identify whether it is under predicting or over predicting since all variations have equal weight.

Equation 1 provides the formula to calculate the MAE.


(1) $$\begin{array}{l} MAE=\frac{1}{N}\overset{N}{\underset{i=1}{\sum }}|SWL_{FOR,i}-SWL_{OBS,i}|, \end{array}$$

where *SWL*_*FOR,i*_ represents the forecast output, SWL_*OBS,i*_ represents the actual output, *N* represents the total number of data points, and *I* represents a single data entry from the data points.

#### Root Mean Squared Error

The RMSE measures the square root of the average squared deviation between the forecast and the actual output, as given in Equation 2. It is used when the error is highly non-linear. The RMSE indicates the amount of errors in the predicted data on average and is a good measure of the prediction accuracy.


(2) $$\begin{array}{l} RMSE=\sqrt{\overset{N}{\underset{i=1}{\sum }}\frac{{\left(SWL_{FOR,i}-SWL_{OBS,i}\right)}^{2}}{N}} \end{array}$$

#### Determination Coefficient

The *R*^2^ metric shows the percentage variation in y explained by x-variables, where x and y signify a set of data. It finds the likelihood of the occurrence of a future event' in the predicted outcome, as given in Equation 3.


(3) $$\begin{array}{r} R^{2}={\left(\frac{n(\sum xy)-(\sum x)(\sum y)}{\sqrt{[n\sum x^{2}-{\left(\sum x\right)}^{2}][n\sum y^{2}-(\sum y{)}^{2}]}}\right)}^{2} \end{array}$$

#### Relative Absolute Error

Relative Absolute Error (RAE) metric gives the ratio of residual or mean error to the forecast error of a naive model. Equation 4, returns a value less than 1, when the proposed model performs better than the naïve model. In Equation 4, “P” stands for the predicted value and “A” for the actual value.


(4) $$\begin{array}{l} RAE=\frac{{\left[\overset{n}{\underset{i=1}{\sum }}{\left(P_{i}-A_{i}\right)}^{2}\right]}^{\frac{1}{2}}}{{\left[\overset{n}{\underset{i=1}{\sum }}A_{i}^{2}\right]}^{\frac{1}{2}}} \end{array}$$

#### Root Relative Squared Error

The Root Relative Squared Error (RRSE) is given as the square root of the relative squared error (RSE). The RSE metric compares the actual forecast error to the forecast error of a naive model. It can be used in models whose errors are measured in different units. As given in Equation 5 and 6, the total squared error is divided by the total squared error of the simple predictor. The simple predictor is just the average of the actual values. The predicted output is “P” and “T” is the target value. Further, the value “i” represents the model and j represents the record. RRSE is given as the square root of the relative squared error, which provides the error in the dimensions of the quantity being predicted, as given in Equation (7). RSE_i_ represents the relative squared error for the model “i”.


(5) $$\begin{array}{lll} RSE_{i} & = & \frac{\overset{n}{\underset{j=1}{\sum }}{\left(P_{ij}-T_{j}\right)}^{2}}{\overset{n}{\underset{j=1}{\sum }}{\left(T_{j}-\bar{T}\right)}^{2}} \end{array}$$

(6) $$\begin{array}{lll} \bar{T} & = & \frac{1}{n}\overset{n}{\underset{j=1}{\sum }}T_{j} \end{array}$$

(7) $$\begin{array}{lll} RRSE & = & \sqrt{RSE_{i}} \end{array}$$

## Results and Discussion

### Results

**Figure 2:**
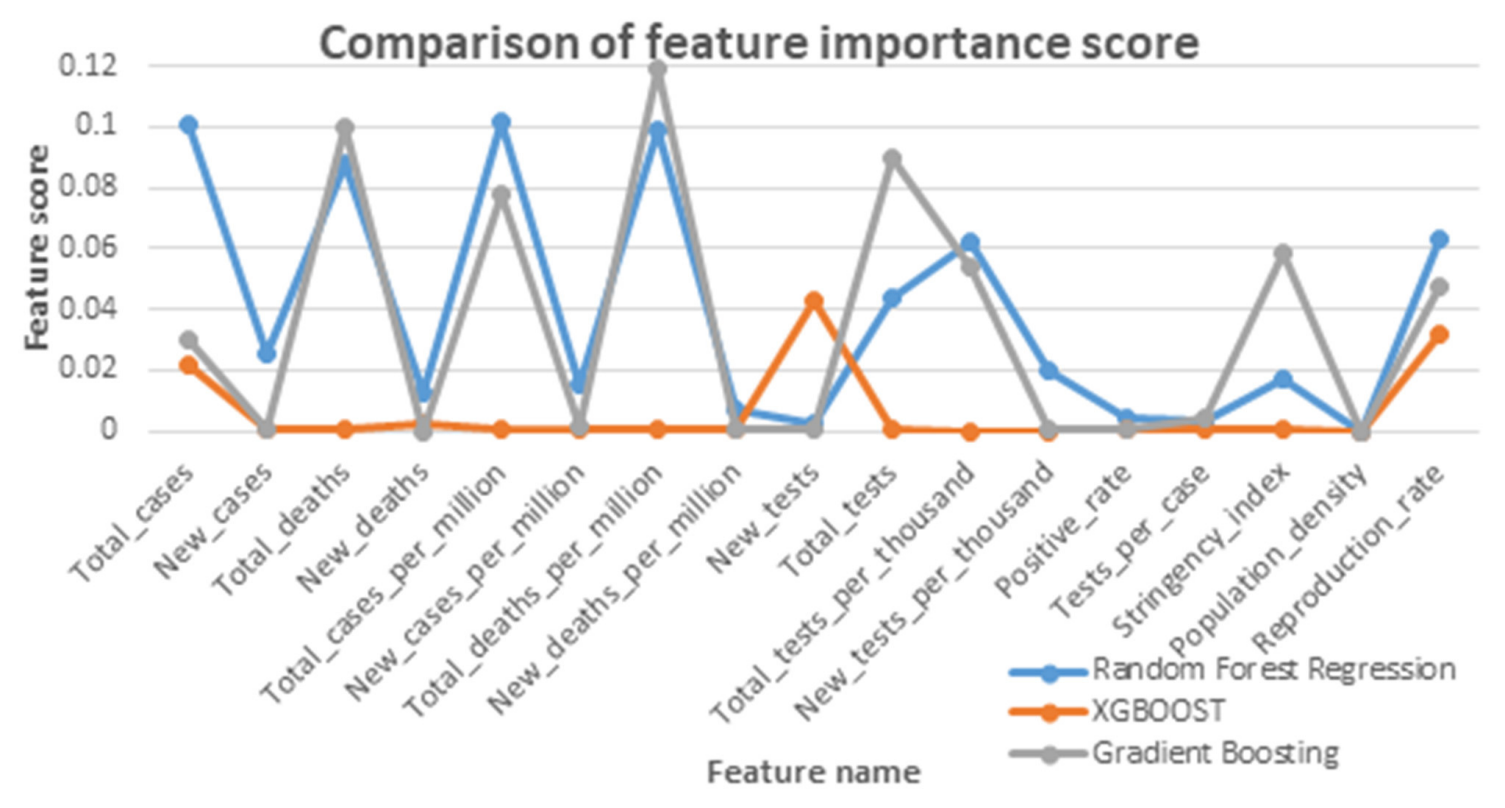
Comparison graph of feature score given by feature selection algorithms.

All experiments were performed using Python's Sci-Kit Library on a Jupyter notebook. The feature importance scores obtained by Random Forest regression, XGBoost, and Gradient Boosting are given in Table 1 and plotted in Figure 2.

**Table 1:** Feature Importance Scores obtained using Random Forest Regression, XGBoost, and Gradient Boosting.

**Features**	**Feature**	**Random forest regression feature importance**	**XGBOOST feature importance**	**Gradient boosting feature importance**
Feature: 0	Total_cases	0.10044	0.92185	0.03024
Feature: 1	New_cases	0.02539	0.00055	0.00058
Feature: 2	Total_deaths	0.08816	0.00011	0.10000
Feature: 3	New_deaths	0.01286	0.00209	0.00000
Feature: 4	Total_cases_per_million	0.10196	0.00013	0.07758
Feature: 5	New_cases_per_million	0.01562	0.00023	0.00176
Feature: 6	Total_deaths_per_million	0.09868	0.00011	0.11893
Feature: 7	New_deaths_per_million	0.00709	0.00021	0.00003
Feature: 8	New_tests	0.00253	0.04245	0.00006
Feature: 9	Total_tests	0.04384	0.00012	0.08950
Feature: 10	Total_tests_per_thousand	0.06210	0.00000	0.05340
Feature: 11	New_tests_per_thousand	0.01958	0.00000	0.00022
Feature: 12	Positive_rate	0.00394	0.00030	0.00090
Feature: 13	Tests_per_case	0.00307	0.00019	0.00376
Feature: 14	Stringency_index	0.01744	0.00020	0.05846
Feature: 15	Population_density	0.00000	0.00000	0.00000

Out of the 16 features, the top seven features affecting the reproduction rate are identified from the obtained feature importance scores. The seven features are Total_cases, New_cases, Total_deaths, Total_cases_per_million, Total_deaths_per_million, Total_tests, Total_tests_per_thousand, and Positive_rate.

Experiments were conducted for the reproduction rate prediction using Random Forest, XGBoost, Gradient Boosting, support-vector regression (SVR), and k**-**nearest neighbor (KNN) regression methods. In addition, the experiments were intended to investigate the impacts of feature selection and hyperparameter tuning. Four experiments were conducted with and without feature selection or hyperparameter tuning. Experiment 1 was conducted using the five regression techniques without feature selection and without parameter tuning. Experiment 2 was conducted with feature selection and without parameter tuning. Experiment 3 was conducted without feature selection and with hyperparameter tuning, and finally, Experiment 4 was conducted with feature selection and with parameter tuning. The reproduction rate prediction was measured using the mean absolute error (MAE), mean squared error (MSE), root mean squared error **(**RMSE), R-Squared, relative absolute error (RAE), and root relative squared error (RRSE). The best, the second-best and the worst results for the particular metrics and experiments are discussed in detail below. The best results obtained for the metrics are given in bold in the Tables 2, 3, **5**, **6**.

**Table 2:** Prediction without Feature Selection and without hyperparameter tuning.

**Sl. No**	**Performance metrics**	**Prediction without feature selection and without hyperparameter tuning**				
		**Random forest regression**	**XGBOOST**	**Gradient boosting**	**KNN**	**SVR**
1	MAE	0.0230122	**0.0189412**	0.02226608	0.0228918	0.0712651
2	MSE	0.0016347	0.0016482	**0.00135535**	0.0018072	0.0064267
3	RMSE	0.0404316	0.0405992	**0.03681510**	0.0425122	0.0801667
4	R-Squared	**0.9792338**	0.9790759	0.97830657	0.9710729	0.8971356
5	RAE	0.1206129	0.3754830	**0.11731593**	0.1306129	0.4754830
6	RRSE	0.1700794	0.1605438	**0.14728681**	0.1700794	0.3207246

**Table 3:** Prediction with Feature Selection and without hyperparameter tuning.

**Performance metrics**	**Prediction with feature selection and without hyperparameter tuning**				
	**Random forest regression**	**XGBOOST**	**Gradient boosting**	**KNN**	**SVR**
MAE	0.02168571	**0.0183910**	0.0225887	0.024706	0.0902574
MSE	**0.00139726**	0.0016258	0.0016091	0.001867	0.0090748
RMSE	**0.03738006**	0.0403218	0.0401140	0.043210	0.0952621
R-Squared	0.97415592	0.9796126	**0.9798852**	0.970115	0.8547498
RAE	0.11583870	**0.0968989**	0.1301729	0.120172	0.3755501
RRSE	**0.15999851**	0.1613162	0.1728719	0.162871	0.1728719

**Figure 3:**
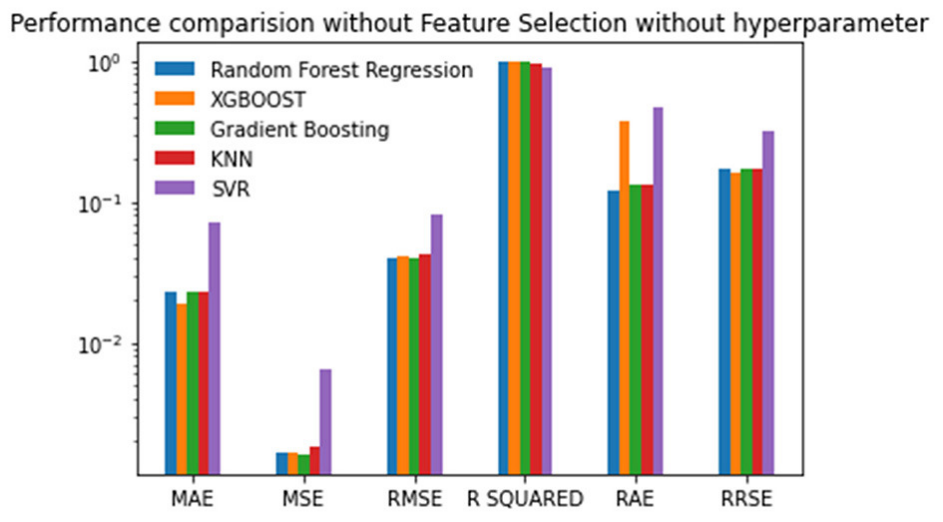
Performance comparison graph of regression techniques without feature selection and without hyperparameter tuning.

The first experiment used all features in the reproduction rate prediction, and each of the regression techniques used the default values for the hyperparameter. The resulting performance metric values are given in Table 2. The Gradient Boosting method performs well with the lowest MSE, RMSE, RAE, and RRSE values and the second-best score for MAE. Random Forest is the next best algorithm with the best R-Squared value and the second-best scores for MSE, RMSE, and RAE. The XGBoost has an average performance. The SVR has the worst scores in all of the performance metrics. The lowest MAE of 0.0189412 was obtained by XGBoost followed by Gradient Boosting with the second-best MAE of 0.02226608. The SVR has the highest MAE of 0.0712651. The minimum MSE, RMSE, RAE, and RRSE values of 0.00135535, 0.036815107, 0.1173159354, and 0.1472868, respectively, are achieved by Gradient Boosting. Random Forest achieves the maximum R-squared value of 0.97923. The obtained metric values are plotted in Figure 3.

**Figure 4:**
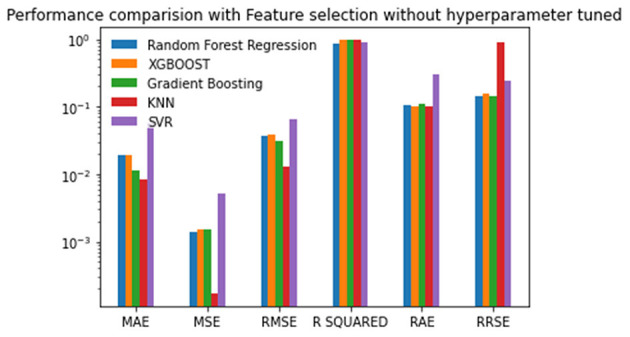
Performance comparison graph of regression techniques with feature selection algorithm and without hyperparameter tuning.

The seven selected features were used in the second experiment, and the prediction process was performed without hyperparameter tuning. There is a reduction in the MAE, MSE, and RMSE values of about 0.01 using Random Forest regression. There is a marginal reduction in the MAE, MSE, and RMSE values using XGBoost and Gradient Boosting for feature selection. The lowest MAE value of 0.018391, RAE value of 0.096898, and the best R-squared value of 0.9796126 is achieved by XGBoost. Random Forest gives the lowest MSE of 0.00139, RMSE of 0.037380, and RRSE of 0.159998. Out of all the algorithms, the SVR produces the highest error rate. The results are given in Table 3 and plotted in Figure 4. The Random Forest regression and XGBoost techniques performed better than the other techniques. The performance of SVR is the worst among the algorithms compared for reproduction rate prediction. In the experiment with feature selection and without hyperparameter tuning, the Random Forest approach has achieved the top performance with three best scores and two second-best scores. The XGBoost has the best scores for the MAE, R-squared, and RAE, and the second-best score for RRSE.

**Figure 5:**
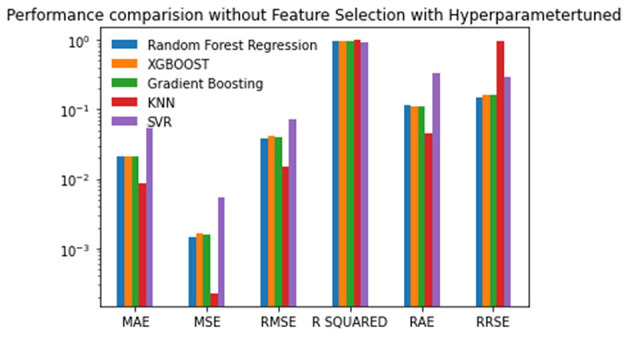
Performance comparison graph of regression techniques without feature selection and with hyperparameter tuning.

Experiment 3 was conducted without feature selection and with hyperparameter tuning. The tuned hyperparameter values are listed in Table 4. The results are good after hyperparameter tuning is performed with the grid search or random search. The results are analyzed based on the best, the second-best, and the last scores. The performance of KNN tops all of the other algorithms when the experiment is performed without feature selection and with hyperparameter tuning. Random Forest is the next best algorithm with the second-best scores for the MSE, RMSE, R-Squared and RRSE. The values are given in Table 5 and plotted in Figure 5.

**Table 4:** Best tuned values of the hyperparameters for the different regression techniques.

**ML algorithms**	**Hyper parameter values**
Random Forest Regression	{“n_estimators”: 800, “min_samples_split”: 2, “min_samples_leaf”: 1, “max_features”: “auto,” “max_depth”: 100, “bootstrap”: True}
KNeighbors Regressor	(n_neighbors = 3, weights = “distance”)
Support Vector Regression	{“C”: 1.5, “epsilon”: 0.1, “gamma”: 1e-07, “kernel”: “linear”}
XGBOOST	{“colsample_bytree”: 0.7, “learning_rate”: 0.1, “max_depth”: 5, “min_child_weight”: 3, “n_estimators”: 500, “objective”: “reg:squarederror,” “subsample”: 0.5}
Gradient Boosting Regression	{“learning_rate”: 0.02, “max_depth”: 10, “n_estimators”: 1,500, “subsample”: 0.5}

**Table 5:** Prediction without Feature Selection and with hyperparameter tuning.

**Performance metrics**	**Prediction without feature selection and with hyperparameter tuning**				
	**Random forest regression**	**XGBOOST**	**Gradient boosting**	**KNN**	**SVR**
MAE	0.021551531	0.020971233	0.021192539	**0.008626385**	0.064263084
MSE	0.001430578	0.001684012	0.001578488	**0.000225718**	0.005462202
RMSE	0.037822976	0.041036714	0.039730185	**0.015023919**	0.073906711
R-Squared	0.977102535	0.973046128	0.974735124	0.996387212	**0.912573368**
RAE	0.113551075	0.110493591	0.111659615	**0.045450843**	0.338590443
RRSE	0.151319084	0.164176344	0.158949287	**0.060106472**	0.295679949

**Figure 6:**
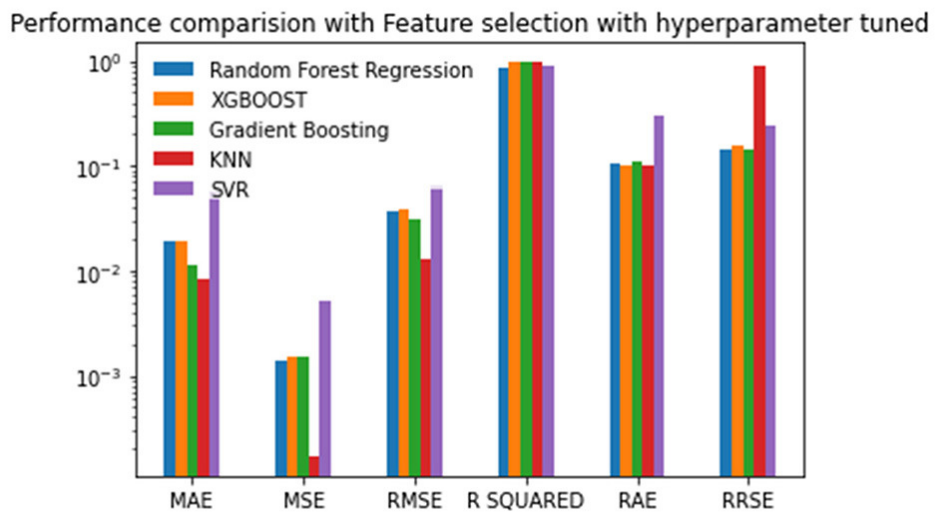
Performance comparison graph of regression techniques with feature selection algorithm and with hyperparameter tuning.

Experiment 4 was conducted with feature selection and with parameter tuning. In this experiment, the Random Forest approach has the best scores for the MSE, RMSE, R-Squared, and RRSE. The XGBOOST has the best scores for the MAE and RAE. Nevertheless, Gradient Boosting has the second-best scores in the MSE, RMSE, R-Squared, and RRSE. The KNN has two second-best scores. Again, the SVR has the worst scores for all of the performance metrics. The values are given in Table 6 and plotted in Figure 6.

**Table 6:** Prediction with Feature Selection and with hyperparameter tuning.

**Performance metrics**	**Prediction with feature selection and with hyperparameter tuning**				
	**Random forest regression**	**XGBOOST**	**Gradient boosting**	**KNN**	**SVR**
MAE	0.020152332	**0.019385962**	0.021180387	0.019439098	0.076691979
MSE	**0.001476030**	0.001715576	0.001683149	0.001706222	0.007260537
RMSE	**0.038419141**	0.041419510	0.041026197	0.04130644	0.085208784
R-Squared	**0.976375028**	0.972540924	0.973059942	0.97269064	0.883789669
RAE	0.106178955	**0.102141089**	0.111595587	0.102421055	0.404076017
RRSE	**0.153704171**	0.165707803	0.164134267	0.165255439	0.340896364

The computation times for the different types of prediction are computed and listed in Table 7. The Random Forest algorithm uses a random search technique for hyperparameter tuning, which requires more time. All of the other algorithms use the grid search technique. The KNN and SVR are able to perform hyperparameter tuning rapidly. XGBOOST and Gradient Boosting regression have moderate running times of around 100 s.

**Table 7:** Running time of algorithms with hyperparameter tuning and prediction.

**ML algorithms**	**Running time including hyperparameter tuning with all features (in seconds)**	**Running time including hyperparameter tuning with selected feature (in seconds)**
Random Forest Regression	492.46183967590330	383.7128930091858
KNeighbors Regressor	0.2534364700317383	0.131617689132690
Support Vector Regression	0.8226490020751953	0.512803316116333
XGBOOST	165.45190143585205	145.1453814506530
Gradient Boosting Regression	120.36712908744812	93.36355471611023

**Figure 7:**
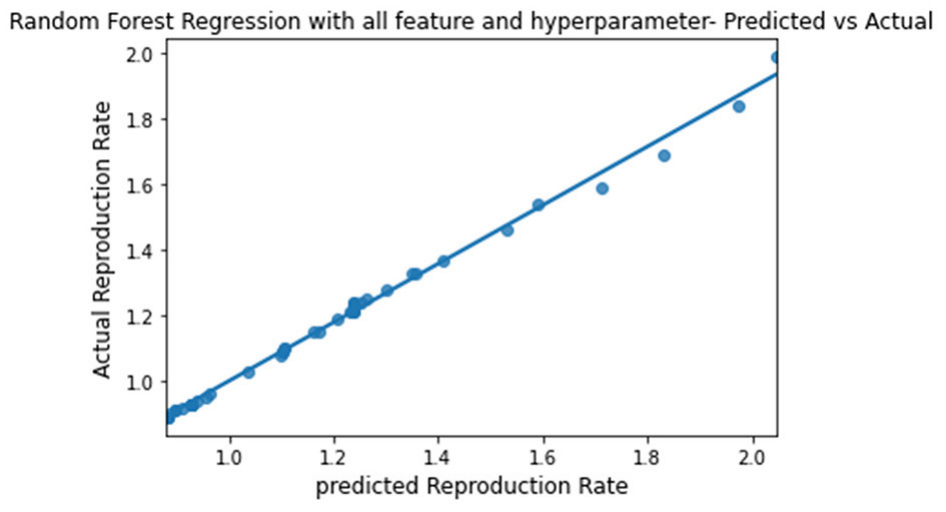
Graph describing the predicted value vs. actual value of Random Forest.

**Figure 8:**
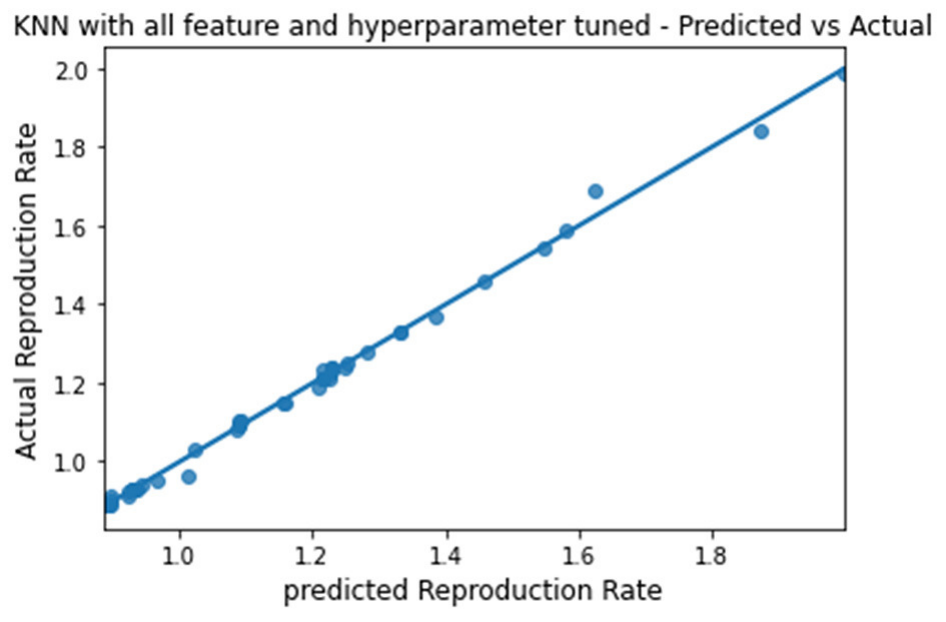
Graph describing the predicted value vs. actual value of KNN.

**Figure 9:**
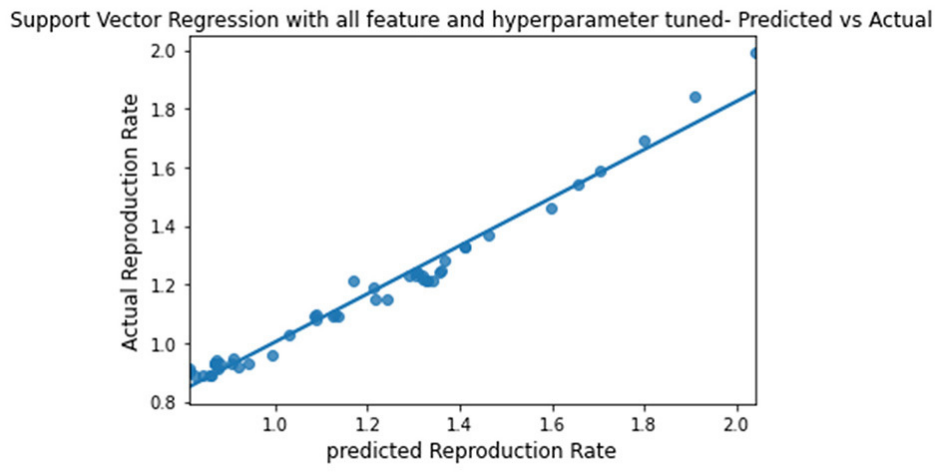
Graph describing the predicted value vs. actual value of SVR.

**Figure 10:**
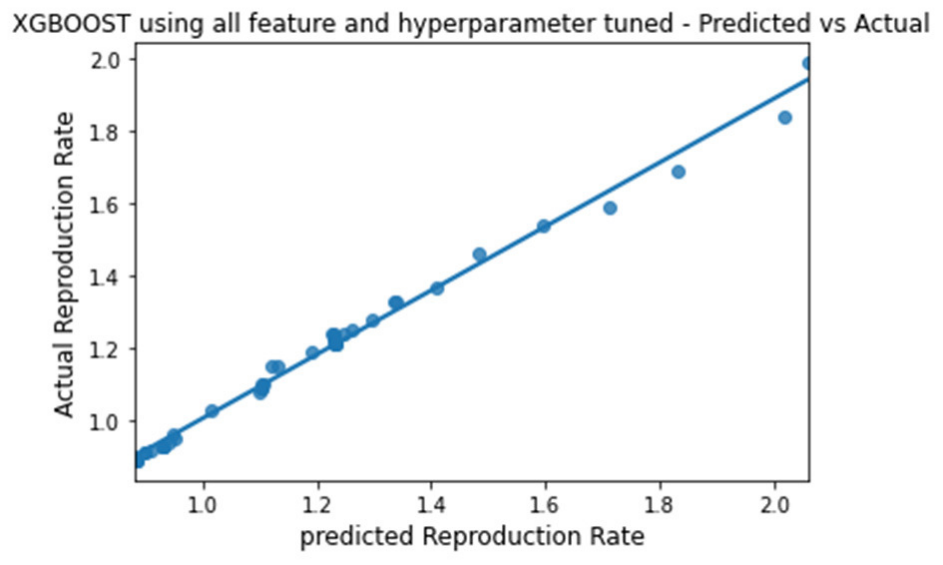
Graph describing the predicted value vs. actual value of XGBoost.

**Figure 11:**
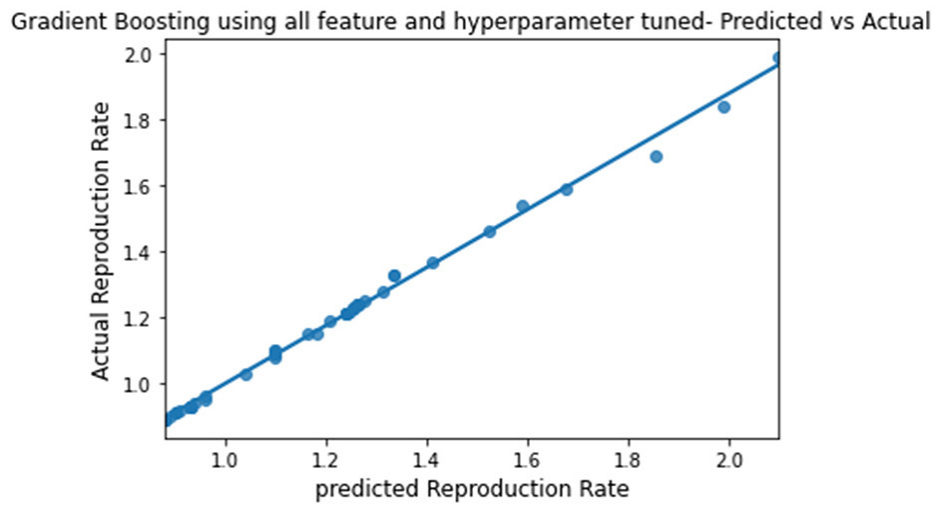
Graph describing the predicted value vs. actual value of Gradient Boosting.

The predicted and actual reproduction rates for Random Forest, KNN, SVR, XGBoost, and Gradient Boosting are, respectively, plotted in Figures 7–11. The graphs show that the predicted values are very close to the actual values.

## Discussion

The major contributions of this paper are the study of features affecting the COVID-19 reproduction rate, as well as the investigation into the effects of feature selection and hyperparameter tuning on the prediction accuracy. Furthermore, prediction accuracy comparisons of the state-of-the-art regression techniques for COVID-19 reproduction rate have also been performed.

The selected features suggest that the total numbers of death and testing also influence the reproduction rate. Instead of depending only on the past value of the predictor variable as cited by Milind et al. (23), our work finds the crucial features affecting the predictor variable. Different regression techniques are used in the prediction and they are used to determine the final reproduction rate. The effectiveness of feature selection in prediction has also been proven. Random forest has achieved the best performance in the accuracy comparison of the state-of-the-art techniques, as has already been proven by Chicco and Jurman (24). In the results obtained by the four experiments, the overall best values of MAE, MSE, RMSE, RAE, RRSE, and R-Squared were all obtained by the KNN approach. Therefore, KNN has obtained the best performance on average, followed by Random Forest and XGBOOST.

## Conclusion and Future Work

Predicting the reproduction rate is crucial, especially when a country has to take preventative measures to protect its citizens from a pandemic. Autoregressive models rely on and work with previous values to forecast future values. Non-linear machine learning regression algorithms have consistently produced the best prediction results in various applications, including the stock exchange, banking, and weather forecasting. Among the many factors involved in the spread of the COVID-19, the prominent factors are identified using Random Forest, Gradient Boosting, and XGBOOST in this work. Random Forest returned the highest importance score for Total_cases_per_million as 0.10196. For XGBOOST, the maximum score was 0.92185 for Total_case, and for Gradient Boosting, the top value of Total_deaths_per_million is 0.1183. Out of 16 features selected for investigation, seven features, namely, Total_cases, New_cases, Total_deaths, Total_cases_per_million, Total_deaths_per_million, Total_tests, Total_tests_per_thousand, and Positive_rate, are found to be prominent in reproduction rate prediction. Furthermore, this work investigated the reproduction rate prediction with non-linear machine learning regression techniques. The experiments were performed using Random Forest, Gradient Boosting, XGBOOST, KNN, and SVR, with and without feature selection and hyperparameter tuning. The results showed a decrease in the prediction error rate with hyperparameter tuning and with all of the features. Overall, the KNN algorithm had obtained the best performance. The study shows that Random Forest obtained the best performance with hyperparameter tuning and selected features. Individual regression techniques are applied in this study. However, the ensemble of regression techniques can be applied to obtain better performances. The regression algorithms obtained improved results with hyperparameter tuning and Gridsearch or Randomsearch methods. There is no remarkable difference in the prediction accuracy of algorithms with and without feature selection algorithms, so there is a need to find out the optimal features related to the reproduction rate.

## Data Availability Statement

The original contributions presented in the study are included in the article/supplementary material, further inquiries can be directed to the corresponding author/s.

## Author Contributions

C-YC and KSr: conceptualization. C-YC: resources, project administration, and funding acquisition. JK and KSu: methodology and software. KSr, SM, and C-YC: validation. KSr, SM, and SC: writing—review and editing. JK: writing—original draft preparation. All authors contributed to the article and approved the submitted version.

## Funding

This research was partially funded by the Intelligent Recognition Industry Service Research Center from The Featured Areas Research Center Program within the framework of the Higher Education Sprout Project by the Ministry of Education (MOE) in Taiwan. Grant number: N/A and the APC were funded by the aforementioned Project.

## Conflict of Interest

The authors declare that the research was conducted in the absence of any commercial or financial relationships that could be construed as a potential conflict of interest.

## Publisher's Note

All claims expressed in this article are solely those of the authors and do not necessarily represent those of their affiliated organizations, or those of the publisher, the editors and the reviewers. Any product that may be evaluated in this article, or claim that may be made by its manufacturer, is not guaranteed or endorsed by the publisher.
